# Role of the Clock Drawing Test in Differential Diagnosis of Alzheimer’s Disease: Clinical Findings in Relation to CSF Biomarkers

**DOI:** 10.3390/ijms27041790

**Published:** 2026-02-13

**Authors:** Aurora Cermelli, Chiara Lombardo, Alberto Mario Chiarandon, Fausto Roveta, Elisa Maria Piella, Virginia Batti, Elisa Rubino, Innocenzo Rainero, Silvia Boschi

**Affiliations:** 1 Aging Brain and Memory Clinic, Department of Neuroscience “Rita Levi Montalcini”, University of Torino, 10126 Turin, Italy; aurora.cermelli@unito.it (A.C.); alberto.chiarandon@unito.it (A.M.C.); fausto.roveta@unito.it (F.R.); elisamaria.piella@unito.it (E.M.P.); virginia.batti@unito.it (V.B.); elisa.rubino@unito.it (E.R.); innocenzo.rainero@unito.it (I.R.); 2 Center for Cognitive Disorders and Dementias (CDCD), Department of Neuroscience and Mental Health, AOU Città della Salute e della Scienza di Torino, 10126 Turin, Italy; c.lombardo@unito.it

**Keywords:** Clock Drawing Test, Alzheimer’s disease, biomarkers, A/T/(N) system, cognitive impairment, diagnostic accuracy, neuropsychological assessment

## Abstract

Alzheimer’s disease (AD) is the most common cause of neurocognitive disorder, and the integration of cognitive assessment with biological markers remains essential for clinical characterization. The Clock Drawing Test (CDT) is a brief and widely used screening tool assessing visuospatial and executive functions, which may reflect underlying neurodegenerative processes. This study investigated the diagnostic performance of the CDT and its association with cerebrospinal fluid (CSF) biomarkers within the A/T/(N) research framework. Ninety-seven patients with mild or major neurocognitive disorder were classified as AD or non-AD according to CSF amyloid-β, phosphorylated tau, and total tau profiles, and compared with 36 healthy participants. All subjects underwent a comprehensive neuropsychological evaluation, including the CDT scored using the quantitative–qualitative method proposed by Rouleau et al. Group comparisons, ROC analyses, and regression models adjusted for age, sex, and education were performed. CDT scores effectively distinguished patients from healthy participants, showing large effect sizes, and modestly differentiated AD from non-AD profiles, particularly on the Hands subscale. Diagnostic accuracy was fair, with adjusted AUC values ranging from 0.65 to 0.75. Lower CDT performance was significantly associated with higher CSF total tau levels, while associations with amyloid-β and phosphorylated tau were not robust after correction. These findings suggest that the CDT is sensitive to cognitive impairment severity and shows limited but meaningful relationships with neurodegenerative biomarkers, supporting its role as a practical complementary tool alongside biological assessment.

## 1. Introduction

Dementia represents one of the most significant clinical conditions of the 21st century worldwide. Alzheimer’s Disease (AD), accounting for up to 80% of all neurocognitive disorder diagnoses, poses a major societal challenge [1,2]. Given the urgent need to validate specific and effective treatments for these disorders, a clear definition of the target population becomes crucial. In this context, research on the biological diagnosis of dementia emerges as a fundamental area for identifying suitable individuals for the application of targeted and personalized therapies.

Increasing evidence on biomarkers highlights their central role in the diagnostic framework of dementias and in predicting the conversion from Mild Cognitive Impairment (MCI) to AD [3]. Biomarkers are measurable indicators of the severity or presence of a disease state. There are several categories of biomarkers relevant to AD, as outlined by the AT(N) framework: Aβ (amyloid beta) biomarkers, indicators of amyloid plaque accumulation, detected through cerebrospinal fluid (CSF) assays measuring Aβ42 levels or amyloid PET imaging; Tau biomarkers, indicators of tau pathology, including phosphorylated tau (p-tau) detected in CSF (e.g., p-tau181, p-tau217, p-tau231) and tau PET imaging for neurofibrillary tangles; neurodegeneration biomarkers, indicators of neuronal injury, such as neurofilament light (NfL) in CSF or plasma, and imaging biomarkers like MRI for structural changes and FDG-PET for metabolic changes. According to the A/T/(N) classification, a neurocognitive disorder is defined by the presence or absence of these biomarkers. This classification system identifies different potential risk profiles within the dementia spectrum, ranging from completely positive A+ T+ N+ to completely negative A−/T−/N−. An A−/T−/ N+, A−/T+/N−, or A−/T+/N+ profile represents a different neuropathologic process from AD, known as “suspected non-Alzheimer’s pathophysiology” (SNAP) [3].

The revised criteria for the diagnosis and staging of AD emphasize a biological definition of the disease, distinguishing it from clinical symptoms that manifest later [4]. The presence of core biomarkers (amyloid and tau) in the absence of symptoms signifies the biological onset of AD, which can be detected through various biomarker assays [4].

While biomarkers provide critical insights into the underlying pathology of AD, they cannot independently diagnose neurocognitive disorders, which require clinical and neuropsychological evaluation to confirm cognitive impairment and functional decline [4]. The biological presence of AD markers, such as amyloid and tau, indicates the disease’s neuropathologic changes but must be interpreted alongside clinical symptoms to establish a diagnosis of dementia [5].

The integration of biomarkers with other clinical information, including comprehensive neuropsychological assessment, improves the characterization and management of dementias [6]. Neuropsychological evaluation provides a detailed characterization of cognitive skills to establish evidence of cognitive impairment, and specifically, offers an evaluation of cognitive domains such as memory, executive functions, language, and visuospatial abilities. The classic clinical profile of AD includes impairments in learning and recall of new information, although recent evidence suggests early involvement of other cognitive domains like executive functions and language [7]. While amyloid biomarkers are becoming more available clinically, the AT(N) framework remains primarily a research tool [3]. Understanding the relationship between cognition and biomarkers is crucial, especially in clinical samples. However, abnormal amyloid accumulation can be observed in individuals without cognitive impairment, limiting the clinical utility of biomarkers alone for prognosis [8]. Adding neuropsychological test scores to biomarker classifications improves predictive accuracy for memory and functional decline [9]. Studies have shown associations between neuropsychological test data and amyloid status, supporting the complementary role of neuropsychology in predicting individual cognitive trajectories. However, further research is needed to understand the concordance between neuropsychological assessments and AD biomarkers obtained in clinical settings [8].

**Figure 1:**
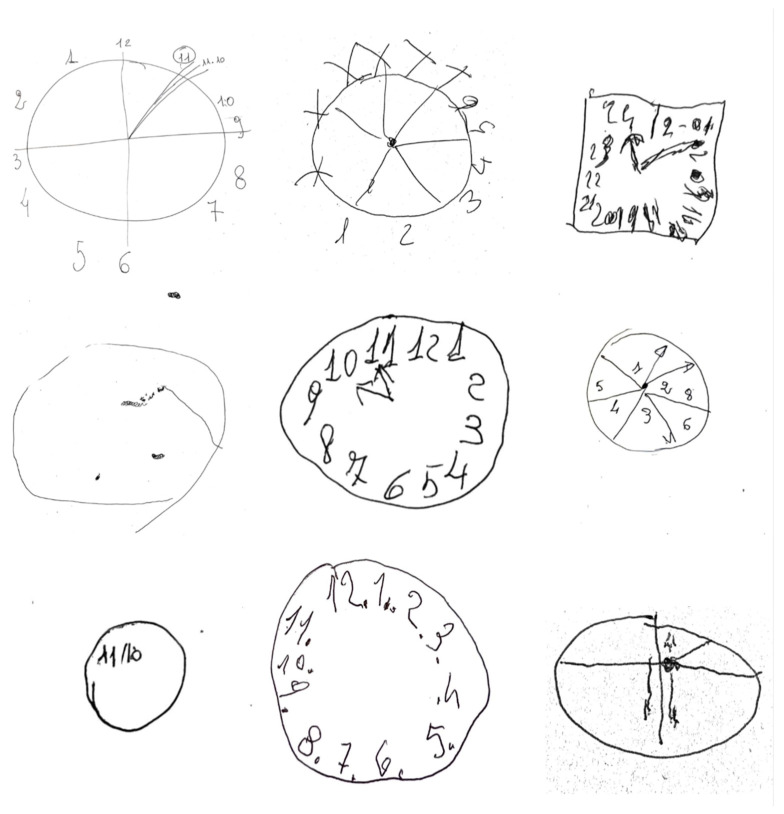
Representative examples of Clock Drawing Test (CDT) performances. Examples of CDT drawings produced by a subset of the recruited participants. The figures illustrate different qualitative and quantitative error patterns commonly observed in patients with neurocognitive disorders.

Among the validated tests, the Clock Drawing Test (CDT) is widely used for screening cognitive impairment [10]. It is a simple neuropsychological instrument characterized by its quick administration, good tolerability by patients, high levels of sensitivity and specificity, low cultural and educational influence, and concurrent and predictive validity [11]. Since the 1980s, its accuracy has been demonstrated in the early detection and prediction of dementia, particularly AD [12,13,14]. Figure 1 shows some examples of clocks drawn by subjects with neurocognitive disorders who were evaluated for this study.

The clock drawing task simultaneously involves various brain areas, including the frontal, temporal, and parietal lobes [15,16,17,18]. Thus, the CDT has the potential to assess multiple cognitive domains, such as executive functions, visuospatial construction and organization, and semantic and abstract knowledge [16,17,18,19]. Different versions of the CDT exist, with variations in the clock contour: the pre-drawn method, in which the clock contour is provided, and the free-drawn method, also known as the “command” condition, which requires drawing a clock from memory on a blank sheet [20,21]. A simpler version involves only drawing the hands on pre-drawn watches with the main characteristics [20].

Another major difference lies in the scoring criteria for the CDT, with several proposed versions [10]. Quantitative assessment systems and qualitative assessment methods are employed. The quantitative approach examines the presence or absence of clock features using a numerical scale. The qualitative CDT score, described by Rouleau et al., identifies the type of error based on its main characteristics [22,23]. Yamamoto and colleagues have shown that a combined quantitative and qualitative scoring method is more accurate in detecting the MCI population compared to a classic and independent approach [14]. Furthermore, a specific type of qualitative miscue, particularly conceptual errors, has been observed to be associated with the progression to dementia from a healthy condition [18,24,25].

Building on previous studies and within an integrated clinical and biologically defined A/T/(N) framework, the current research aims to investigate whether the CDT is a sensitive tool for detecting correlations between the type of error committed in the CDT and the nature of cognitive impairment. Specifically, it aims to determine if the CDT is valuable in differentiating the AD spectrum from non-AD, when patients are investigated according to the A/T/(N) research framework [18,23,26,27].

## 2. Results

### 2.1. Demographic and Clinical Characteristics

A total of 133 participants were included: 97 patients with neurocognitive disorders (47 classified as AD and 50 as non-AD) and 36 healthy comparison participants. Demographic and clinical characteristics are summarized in Table 1.

The three groups differed significantly in age (*p* = 0.003), with healthy participants being younger than both patient groups. Education also differed across groups (*p* = 0.009), as expected, with higher educational attainment in the healthy group. No significant sex differences were observed (χ^2^ = 1.28, *p* = 0.53).

Global cognitive performance (MMSE and MoCA) was lower in both patient groups compared to healthy participants (all *p* < 0.001). Within the clinical sample, the AD group scored lower than the non-AD group on both measures (*p* = 0.018 and *p* = 0.008, respectively).

### 2.2. CDT Quantitative and Qualitative Performance

#### 2.2.1. Patients vs. Healthy Participants

Both the classical CDT total score and the global CDT index were significantly lower in patients compared to healthy participants (both *p* < 0.001), with large effect sizes (Cohen’s d = 1.31 for the classical score and 1.37 for the global index). Subscale analysis showed robust differences in the Numbers (*p* < 0.001) and Hands (*p* < 0.001) components, while the Contour subscale showed a smaller effect (*p* = 0.040).

Qualitative errors were more frequent in patients than in healthy participants for all error categories (all *p* < 0.01). The largest group differences were found for conceptual errors (*p* < 0.001) and stimulus-bound responses (*p* < 0.001).

#### 2.2.2. AD vs. Non-AD Groups

CDT performance was compared using the classical quantitative scoring method (0–15) and a global score integrating quantitative and qualitative components (0–10), as described in the Methods section.

**Figure 2:**
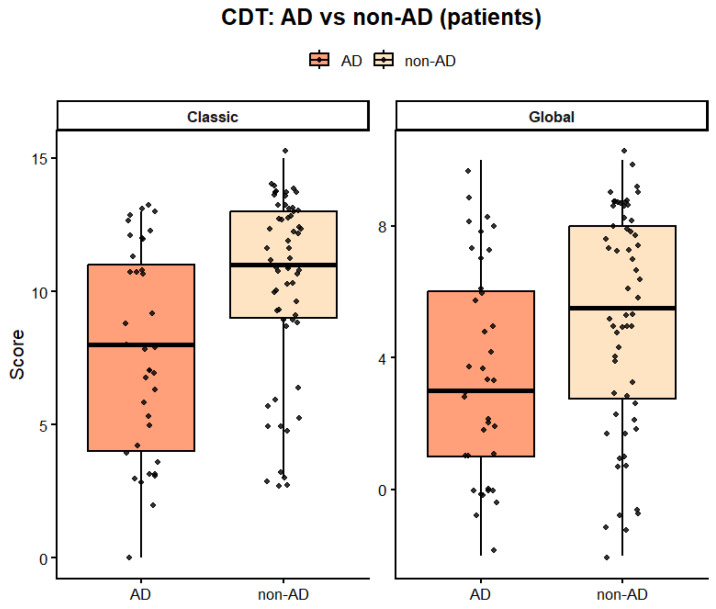
Comparison of classical and global CDT scores in AD and non-AD patients. Distribution of CDT scores in AD and non-AD patients. Boxplots represent the classical CDT total score, based on a quantitative scoring scheme, and the global CDT index, which combines quantitative and qualitative components of performance. Individual data points are shown. Boxes indicate the median and interquartile range, with whiskers representing the data range.

Within the clinical sample, AD patients exhibited significantly lower CDT performance than non-AD patients. The distribution of scores obtained with the two scoring approaches is shown in Figure 2.

For the classical CDT score, mean values were 7.12 ± 1.82 for AD and 8.25 ± 1.44 for non-AD (*p* < 0.001).

For the global CDT index, mean values were 5.47 ± 2.10 for AD and 6.68 ± 1.95 for non-AD (*p* = 0.003). The magnitude of these differences was moderate, as indicated by Cohen’s d = 0.70. 

The Hands subscale was the most discriminant (*p* < 0.001), while Contour and Numbers showed smaller but consistent effects (*p* = 0.041 and *p* = 0.027, respectively).

Regarding qualitative errors, AD patients committed more conceptual and stimulus-bound mistakes than non-AD patients (*p* = 0.012 and *p* = 0.008, respectively), but the groups did not differ on spatial or planning errors (all *p* > 0.10).

After adjustment for age, education, and sex using covariate-adjusted models, the group effect on CDT total and global scores remained significant (*p* < 0.01), confirming that these results were not attributable to demographic confounders.

Table 2 outlines the means and frequencies of the eleven items of the CDT in the three study groups.

### 2.3. Diagnostic Accuracy

Receiver operating characteristic (ROC) analyses were conducted to assess the diagnostic performance of CDT indices for distinguishing AD from non-AD patients.

The classical CDT yielded an AUC of 0.69 (95% CI 0.58–0.79, *p* = 0.002), whereas the global CDT showed a slightly lower AUC of 0.65 (95% CI 0.55–0.76, *p* = 0.008). After adjusting logistic regression models for age, education, and sex, both indices improved in predictive accuracy (AUC = 0.75 and 0.71, respectively).

Youden’s index identified optimal CDT cut-off values of ≤7 for the classical score and ≤5 for the global index, corresponding to sensitivities of 0.70–0.72 and specificities of 0.67–0.69.

### 2.4. Associations Between CDT and CSF Biomarkers

Lower CDT scores were significantly associated with higher total tau (r_s_ = −0.28, *p* = 0.015) and higher phosphorylated tau (r_s_ = −0.22, *p* = 0.047). The correlation with Aβ_42_ was positive but not statistically significant (r_s_ = 0.18, *p* = 0.09).

In linear regression models adjusted for age, education, and sex, total tau (tTau) remained an independent predictor of CDT performance when using the classical CDT score (β = −0.29, *p* = 0.015). A similar association was observed for the global CDT index (β = −0.23, *p* = 0.049), while phosphorylated tau and Aβ_42_ did not reach statistical significance in either model. Adding CDT indices to models including CSF biomarkers resulted in only minimal changes in AUC values (ΔAUC = +0.02), suggesting limited incremental predictive value beyond biomarkers alone.

**Figure 3:**
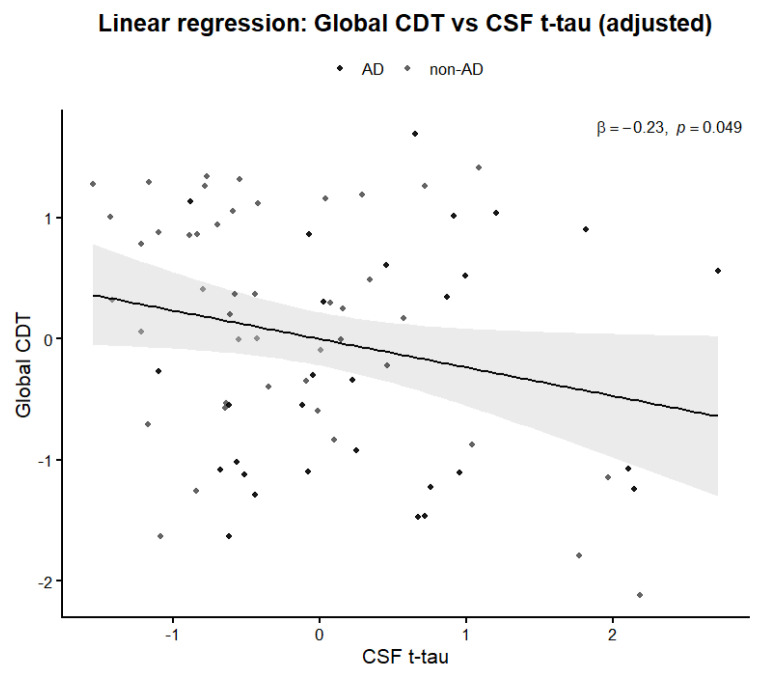
Relationship between global CDT performance and CSF tTau (tTau) levels. Association between global CDT score and CSF tTau levels in patients with neurocognitive disorders. The solid line represents the fitted regression line, and the shaded area indicates the 95% confidence interval. Lower CDT scores tend to be associated with higher tTau concentrations.

**Figure 4:**
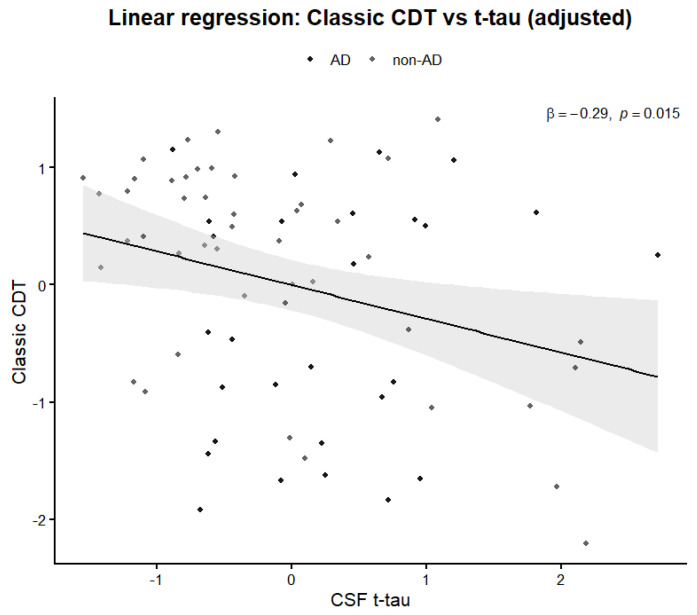
Relationship between classic CDT performance and CSF tTau levels. Association between the classic CDT score and CSF tTau levels in patients with neurocognitive disorders. The solid line represents the fitted regression line, and the shaded area indicates the 95% confidence interval. Lower CDT scores tend to be associated with higher tTau concentrations.

The relationship between classic and global CDT performance and CSF total tau levels is illustrated in Figure 3 and Figure 4.

## 3. Discussion

This study explored the utility of the CDT in distinguishing between biomarker-defined AD and non-AD neurocognitive disorders and examined its association with CSF biomarkers within the A/T/(N) classification framework [3]. CDT, as described in the introduction, is a tool widely used in clinical practice for assessing cognitive deficits in individuals with suspected AD or other forms of neurodegenerative disorders. In this study, however, we aim to explore an alternative and more comprehensive way of interpreting CDT results, investigating both quantitative and qualitative aspects, and to explore whether there is a significant difference between the scoring of AD patients versus those non-AD and the responses of the control group. As mentioned before, the CDT is a very useful neuropsychological tool because it is rapid to administer, non-invasive, and can be used as a screening tool to detect visuospatial, praxis, memory, and executive deficits. We propose this scoring method to quantify individual neuropsychological subdomains and to detect specific symptoms that may be useful in discriminating between AD and non-AD patients. The global CDT score is the summarized variable, comprising the following components: quantitative scoring (Contour, Numbers, and Hands) and qualitative errors (stimulus-bound response, conceptual deficits, perseveration, neglect of the left hemispace, nonspecific spatial errors, numbers written outside the clock, and counterclockwise number arrangement).

To date, this study has been among the first to examine CDT performance in relation to cognitive impairment profiles within a biologically defined A/T/(N) framework, moving beyond diagnostic classifications based exclusively on clinical presentation.

Consistent with prior evidence, the CDT effectively differentiated cognitively impaired patients from healthy participants, showing large effect sizes across both classical and global scoring systems. These findings confirm the CDT’s robustness as a global cognitive screening measure that integrates multiple functions—including executive, visuospatial, and semantic components—frequently affected in neurodegenerative conditions.

Within the clinical cohort, CDT performance modestly differentiated AD from non-AD patients, yielding AUC values between 0.65 and 0.75 after covariate adjustment. According to conventional interpretation, these results indicate poor-to-fair discrimination. Although modest, this degree of accuracy aligns with expectations for a brief, behavior-based instrument and supports its potential as an accessible adjunct to biomarker evaluation. Notably, the Hands subscale demonstrated the greatest sensitivity to AD-related impairment, consistent with the view that hand placement reflects executive–visuospatial integration and temporal sequencing—functions dependent on parietal–frontal networks often disrupted in AD [28].

The qualitative analysis further underscored that conceptual and stimulus-bound errors were more frequent in AD patients, aligning with prior reports linking these errors to semantic and inhibitory control deficits typical of Alzheimer’s pathology [29]. In contrast, spatial and planning errors, which may reflect more general executive dysfunction, did not discriminate between the groups. This pattern suggests that CDT errors may reflect specific cognitive signatures associated with distinct neurodegenerative processes.

The exploratory correlations between CDT scores and CSF biomarkers revealed a weak but consistent relationship with total tau, while associations with Aβ_42_ and pTau were non-significant after correction for multiple testing. Although these effects were modest, their direction is biologically coherent: lower CDT performance was associated with a higher neurodegenerative burden. Consistent with current models of the Alzheimer’s disease continuum, tau-related neurodegeneration is increasingly recognized as a key driver of the transition from preclinical to symptomatic stages, which explains the closer association observed between CDT performance and CSF total tau. CDT performance appears to be more closely related to the cognitive expression of tau-related neurodegeneration rather than to quantitative measures of tau pathology. Literature evidence underscores tau protein’s role as a fundamental component of neurofibrillary tangles, primarily localized in axons, dendrites, and cell bodies. Elevated CSF levels of t-tau have been observed in patients with MCI and AD compared to healthy subjects, detectable even in early AD stages and remaining stable over time [30]. The observed inverse correlation between the CDT score and total tau levels in our study suggests that the CDT might also be sensitive to neurodegeneration in conditions beyond AD. This association hints at the potential utility of the CDT as a screening tool for assessing neurodegeneration across a broader spectrum of neurodegenerative diseases. However, this finding does not imply disease specificity. Consistent with the modest AD vs. non-AD discrimination observed in our analyses, CDT performance appears to reflect the cognitive expression of neurodegeneration rather than Alzheimer’s disease-specific pathology. Further investigations are necessary to fully explore the CDT’s potential as a complementary diagnostic tool in evaluating neurodegeneration and neurodegenerative diseases.

When comparing our findings with those of Bruno et al. [31], who examined the association between the recency ratio (Rr) from Rey’s AVLT and CSF biomarkers, several parallels emerge. Both studies highlight the importance of cognitive tests in relation to CSF tau levels. Our study, while focusing on the CDT, similarly found a significant association with CSF t-tau levels. The consistency in findings across these studies supports the hypothesis that specific cognitive test scores are reliable indicators of tau pathology. Bruno et al. [31] posited that Rr’s sensitivity to tau pathology is likely due to its reflection of medial temporal lobe function, which is significantly impacted in tauopathies. This aligns with our observation of the CDT’s sensitivity to t-tau, as the CDT evaluates visuospatial and executive functions, areas often affected early in the neurodegenerative process.

While this study contributes valuable insights, it also has some limitations. Firstly, the CDT serves as a clinical tool, necessitating complementation with other biological investigations for the diagnosis of neurodegenerative disorders. Furthermore, its efficacy is heightened when integrated with additional neuropsychological assessments to comprehensively quantify cognitive impairments. Other variables should also be considered, specifically neuropsychiatric comorbidities, which are known to influence cognitive performance. Additionally, the dementia stage was established through clinical judgment rather than a standardized staging scale (e.g., CDR). The absence of a formal staging measure may have limited more precise control for disease severity across groups and should be addressed in future studies.

The utility of this tool resides in its ease of administration, patient-friendly nature, and minimal time commitment. However, the identified CDT features need further exploration in larger cohorts to validate their specificity and sensitivity. Expanding the scope, future research could benefit from larger sample sizes, particularly within the non-AD group, allowing for better distinctions among subtypes such as dementia with Lewy bodies, frontotemporal dementia, vascular dementia, or mixed dementia.

Furthermore, future research should also integrate CDT with other biomarkers to investigate the predictive value of the tool when combined with other non-invasive diagnostic instruments, such as plasma biomarkers, to facilitate large-scale disease screening in the population.

## 4. Materials and Methods

### 4.1. Participants

Ninety-seven patients diagnosed with mild or major neurocognitive disorder according to DSM-5 criteria were consecutively recruited between 2023 and 2024 at the Memory Clinic of the Department of Neuroscience, University of Torino, Italy. Each participant underwent a standardized diagnostic protocol, including a neurological examination, neuropsychological evaluation, brain imaging, and CSF biomarker analysis.

Patients were categorized into AD and non-AD groups according to the A/T/(N) research framework. CSF biomarkers included amyloid-β_42_ (Aβ_42_), phosphorylated tau (pTau), and total tau (tTau). Locally validated cut-off values, harmonized with BIOMARKAPD recommendations and the Italian AD Network, were applied: Aβ_42_ < 600 pg/mL, pTau > 61 pg/mL, and tTau > 400 pg/mL. Individuals with an amyloid-positive profile (A+/T+/(N)+ or A+/T+/(N)−) were classified as AD, while those with non-amyloid or mixed profiles (e.g., A−/T−/(N)+, A−/T+/(N)−) were classified as non-AD.

The non-AD group included patients with a clinical diagnosis of dementia with Lewy bodies, frontotemporal dementia, vascular dementia, and mixed dementia.

A healthy comparison group of 36 participants was also included. They were community volunteers and caregivers who underwent clinical and neuropsychological screening to exclude neurological, psychiatric, or cognitive disorders. None was taking psychoactive medication, and all performed within normal limits on global screening tests. The term healthy comparison group is used throughout to reflect their non-experimental, observational role.

The study was approved by the local Ethics Committee (Approval Code: 001863). All participants provided written informed consent for the use of their anonymized data for research purposes, in accordance with the Declaration of Helsinki.

### 4.2. Neuropsychological Assessment

All participants underwent a comprehensive neuropsychological battery assessing the main cognitive domains: memory, attention, executive function, logical–deductive reasoning, praxis, visuospatial ability, and language. The battery included the Mini-Mental State Examination (MMSE), Montreal Cognitive Assessment (MoCA), Frontal Assessment Battery (FAB), Trail Making Test A and B (TMT-A/B), Rey Auditory Verbal Learning Test (immediate and delayed recall), Phonemic and Semantic Verbal Fluency, Rey–Osterrieth Complex Figure (copy and delayed recall), and Raven’s Colored Progressive Matrices (CPM-47).

For the present study, the MoCA and CDT were the primary measures of interest. It is important to note that the MoCA includes a clock drawing item, which may partially contribute to the observed correlation between the CDT and MoCA total scores.

Dementia severity was determined clinically as part of the comprehensive neurological and neuropsychological evaluation performed at the memory clinic.

### 4.3. CSF Biomarkers

CSF samples were collected by lumbar puncture in the morning after overnight fasting. Analyses were performed using commercially available ELISA kits (Fujirebio^®^, Ghent, Belgium) to quantify Aβ_42_, pTau, and tTau concentrations. The cut-off values applied for biomarker positivity were derived from locally validated, inter-laboratory harmonized reference intervals consistent with BIOMARKAPD guidelines. Patients lacking complete CSF data were excluded from the biomarker analyses.

The Aβ42/Aβ40 ratio was not available for the entire cohort, as it was not routinely measured in our laboratory during the CSF collection period. Therefore, amyloid status was determined using CSF Aβ42 cut-offs to ensure consistency across participants.

### 4.4. Clock Drawing Test

Regarding the analysis of CDT performance, the same methodology was employed for both test administration and scoring. Specifically, participants were instructed as follows: “Please draw the face of a clock, include all the numbers, and set the clock hands to 11 and 10.”

Subsequently, each clock drawing was assigned a quantitative score ranging from 0 to 10, a qualitative score ranging from 0 to 8, and an overall score, according to the scheme proposed by Rouleau et al. [23]. The global Clock Drawing Test (CDT) score was calculated by subtracting the qualitative error score from the quantitative score. This comprehensive score takes into consideration not only the presence and accuracy of clock features but also the number of different error types made and the strategies utilized in constructing the clock.

In Table 3, the criteria for assigning the quantitative score are specified, and in Table 4, the criteria for the qualitative score are provided.

### 4.5. Statistical Analysis

All statistical analyses were performed using R (v4.5.1). Continuous variables are presented as mean ± standard deviation (SD) and categorical variables as frequency (%).

Group comparisons (patients vs. healthy participants; AD vs. non-AD) were evaluated using independent-sample *t*-tests; for CDT outcomes, additional analyses of covariance (ANCOVA) adjusted for age, education, and sex were performed.

Effect sizes were expressed as Cohen’s d. Categorical variables were analyzed using χ^2^ tests or Fisher’s exact tests, as appropriate. Associations between CDT performance and CSF biomarkers (Aβ_42_, pTau, tTau) were explored using Spearman correlations and linear regression models adjusted for age, sex, and education.

Diagnostic discrimination between AD and non-AD was assessed through ROC curve analyses. Youden’s index identified optimal sensitivity–specificity thresholds. Binomial logistic regression models were fitted including demographic covariates (age, education, sex) and each CTD index standardized as a z-score. Model performance was evaluated using AUC and the area under the precision-recall curve (AUPRC).

Given the moderate sample size and unequal group distribution, findings were interpreted cautiously with attention to statistical power. Statistical significance was defined as *p* < 0.05 (two-tailed).

For all continuous variables presented in Table 3, both mean ± SD and minimum–maximum ranges are reported to provide a comprehensive description of sample distribution.

## 5. Conclusions

The CDT remains a simple yet powerful tool for assessing cognitive decline in neurodegenerative disorders. While it cannot replace biomarker testing, it offers valuable complementary information when integrated with biological data. In this study, the CDT effectively differentiated cognitively impaired patients from healthy individuals and modestly distinguished AD from non-AD profiles, with performance reflecting the functional cognitive consequences of tau-associated neurodegeneration.

These findings reinforce the role of multimodal assessment in dementia diagnostics and highlight the potential of behavioral markers to complement molecular characterization within the precision medicine framework.

## Figures and Tables

**Table 1:** Demographic, clinical, and Cerebrospinal Fluid (CSF) biomarker characteristics of the study sample. Demographic and clinical characteristics of patients with Alzheimer’s (AD), non-AD neurocognitive disorders, and healthy control participants. Data are presented as mean (standard deviation) for continuous variables and as counts for categorical variables. CSF biomarker values are reported for the patient groups only. MMSE, Mini-Mental State Examination; CSF, cerebrospinal fluid; Aβ_42_, amyloid beta 42; tTau, total tau; pTau, phosphorylated tau.

*Group*	Age Mean (SD)	Education Mean (SD)	Male/ Female	MMSE Mean (SD)	CSF Aβ_42_ Mean (SD)	CSF t-Tau Mean (SD)	CSF p-Tau Mean (SD)
AD	68.30 (7098)	11.92 (4462)	12/25	21.00 (5568)	408 (158)	99.4 (61.0)	659 (356)
Non-AD	68.75 (10,151)	10.80 (4368)	32/28	23.73 (5054)	677 (365)	71.5 (72.1)	451 (347)
Controls	63.36 (8771)	14.39 (3358)	9/27	29.00 (1000)	-	-	-

**Table 2:** Quantitative and qualitative CDT performance across study groups. Quantitative and qualitative CDT performance in patients with AD, non-AD neurocognitive disorders, and healthy control participants. Mean values are reported for quantitative CDT subcomponents (Contour, Numbers, and Hands). Qualitative error frequencies are expressed as the number of subjects presenting each error over the total number of participants in each group (n/N).

*Group*	Contour (Mean)	Numbers (Mean)	Hands (Mean)	*Stimulus-Bound* (n)	*Conceptual Deficit* (n)	*Perseveration* (n)	*Neglect* (n)	*Planning* (n)	*Nonspecific Spatial Error* (n)	*Numbers Written Outside the Clock* (n)	*Counterclockwise Number Arrangement* (n)
*AD*	1.78	2.00	1.84	18/37	26/37	7/37	0/37	3/37	13/37	7/37	2/37
*Non-AD*	1.60	2.61	2.65	22/60	41/60	5/60	2/60	4/60	12/60	7/60	1/60
*Controls*	1.83	3.69	3.50	3/36	6/36	0/36	0/36	0/36	2/36	7/36	0/36

**Table 3:** Quantitative scoring criteria for the CDT. Criteria for the quantitative scoring of the CDT according to the method proposed by Rouleau et al. The total quantitative score ranges from 0 to 10 and is derived from three components: Contour, Numbers, and Clock Hands. Each component is scored based on the presence and accuracy of specific features, as detailed in the table.

Quantitative Score (0–10)
Contour (0–2) • Acceptable contour (0–1) • Contour not too small or intermittently reproduced (0–1)
Numbers (0–4) • Numbers 1–12 without omissions or extra insertions (0–1) • Numbers all in the correct positions (0–1) • Numbers all Arabic or all Roman numerals (0–1) • Numbers in the correct order (0–1)
Clock Hands (0–4) • Clock has two hands or marks (0–1) • Hour and minutes are indicated in some way (0–1) • Proportions of the clock hands are correct (0–1) • Hands are connected to form the center of the clock (0–1)

**Table 4:** Qualitative error classification for the CDT. Classification of qualitative errors in the CDT according to Rouleau et al. The qualitative score ranges from 0 to 8 and reflects the presence of specific errors: stimulus-bound response, conceptual deficit, perseveration, neglect of the left hemispace, planning deficit, nonspecific spatial error, numbers written outside the clock, and counterclockwise number arrangement.

Qualitative Score—Errors (Total 0–8)
1. Stimulus-bound response. The tendency of the drawing to be dominated or guided by a single stimulus. Three types of stimulus-bound errors can occur: (a) Clock hands set to 11 minus ten instead of 11 and 10; (b) Hour written next to 11 or between 10 and 11 on the clock; (c) Clock hands absent or pointing to “10” and/or “11”. This type of error is also considered a conceptual error.
2. Conceptual deficit. Error reflecting a loss or deficit in accessing semantic memory (knowledge of the characteristics, meaning, and attributes of a clock). This deficit may also lead to a misrepresentation of the clock itself (a dial without numbers or inappropriate use of numbers) and the hour on the dial (hands absent or inadequately represented, or hour written on the clock).
3. Perseveration. Construction or repetition of the test without an appropriate stimulus. In clock drawing, this error is equivalent to depicting more than two hands and abnormal continuation of the numbers (writing beyond “12”).
4. Neglect of the left hemispace. All elements of the clock are written in the right half of the dial. The possible neglect of the right hemispace was also evaluated, but this type of error was never observed.
5. Planning deficit. This error is represented by the presence of spaces before 12, 3, 6, or 9, depending on the drawing strategy used.
6. Nonspecific spatial error. Deficit in the spatial arrangement of numbers without a specific pattern of spatial disorganization.
7. Numbers written outside the clock. Numbers written around the perimeter of the circle or on the circle itself.
8. Counterclockwise number arrangement. Arrangement of numbers with “12” at the top of the dial and continuing counterclockwise.

## Data Availability

The data presented in this study are not publicly available due to privacy and ethical restrictions. Data are available from the corresponding author upon reasonable request, subject to approval by the Ethics Committee and compliance with applicable regulations.

## Abbreviations

The following abbreviations are used in this manuscript:

AD	Alzheimer’s Disease
ANCOVA	Analysis of Covariance
AUC	Area Under the Curve
AUPRC	Area Under the Precision–Recall Curve
AT(N)	Amyloid/Tau/Neurodegeneration Framework
Aβ_42_	Amyloid-Beta 42
BIOMARKAPD	Biomarkers for Alzheimer’s Disease
CDT	Clock Drawing Test
CI	Confidence Interval
CPM-47	Raven’s Colored Progressive Matrices (47 items)
CSF	Cerebrospinal Fluid
DSM-5	Diagnostic and Statistical Manual of Mental Disorders, Fifth Edition
ELISA	Enzyme-Linked Immunosorbent Assay
FAB	Frontal Assessment Battery
FDR	False Discovery Rate
FDG-PET	Fluorodeoxyglucose Positron Emission Tomography
MCI	Mild Cognitive Impairment
MMSE	Mini-Mental State Examination
MoCA	Montreal Cognitive Assessment
MRI	Magnetic Resonance Imaging
NfL	Neurofilament Light
OR	Odds Ratio
PET	Positron Emission Tomography
pTau	Phosphorylated Tau
RAVLT	Rey Auditory Verbal Learning Test
ROC	Receiver Operating Characteristic
SD	Standard Deviation
SNAP	Suspected Non-Alzheimer’s Pathophysiology
tTau	Total Tau
TMT-A/B	Trail Making Test Parts A and B
